# The Role of Solvent-Accessible Leu-208 of Cold-Active *Pseudomonas fluorescens* Strain AMS8 Lipase in Interfacial Activation, Substrate Accessibility and Low-Molecular Weight Esterification in the Presence of Toluene

**DOI:** 10.3390/molecules22081312

**Published:** 2017-08-12

**Authors:** Norhayati Yaacob, Nor Hafizah Ahmad Kamarudin, Adam Thean Chor Leow, Abu Bakar Salleh, Raja Noor Zaliha Raja Abd Rahman, Mohd Shukuri Mohamad Ali

**Affiliations:** 1 Enzyme Technology/Molecular Biomedicine Laboratory, Enzyme and Microbial Technology Research Centre, Institute of Bioscience, Universiti Putra Malaysia, 43400 Serdang, Malaysia; norhayati86ny@yahoo.com; 2 Enzyme and Microbial Technology Research Centre, Faculty of Biotechnology and Biomolecular Sciences, University Putra Malaysia, 43400 Serdang, Malaysia; hafizah_kamar@upm.edu.my; 3 Enzyme and Microbial Technology Research Centre, Department of Cell and Molecule Biology, Faculty of Biotechnology and Biomolecular Sciences, Universiti Putra Malaysia, 43400 Serdang, Malaysia; adamleow@upm.edu.my; 4 Enzyme and Microbial Technology Research Centre, Department of Biochemistry, Faculty of Biotechnology and Biomolecular Sciences, Universiti Putra Malaysia, 43400 Serdang, Malaysia; abubakar@upm.edu.my; 5 Enzyme and Microbial Technology Research Centre, Department of Microbiology, Faculty of Biotechnology and Biomolecular Sciences, Universiti Putra Malaysia, 43400 Serdang, Malaysia; rnzaliha@upm.edu.my

**Keywords:** molecular dynamics, cold-active lipase, substrate-accessibility, interfacial activation, hydrolysis, esterification

## Abstract

The alkaline cold-active lipase from *Pseudomonas fluorescens* AMS8 undergoes major structural changes when reacted with hydrophobic organic solvents. In toluene, the AMS8 lipase catalytic region is exposed by the moving hydrophobic lid 2 (Glu-148 to Gly-167). Solvent-accessible surface area analysis revealed that Leu-208, which is located next to the nucleophilic Ser-207 has a focal function in influencing substrate accessibility and flexibility of the catalytic pocket. Based on molecular dynamic simulations, it was found that Leu-208 strongly facilitates the lid 2 opening via its side-chain. The K_M_ and K_cat_/K_M_ of L208A mutant were substrate dependent as it preferred a smaller-chain ester (pNP-caprylate) as compared to medium (pNP-laurate) or long-chain (pNP-palmitate) esters. In esterification of ethyl hexanoate, L208A promotes a higher ester conversion rate at 20 °C but not at 30 °C, as a 27% decline was observed. Interestingly, the wild-type (WT) lipase’s conversion rate was found to increase with a higher temperature. WT lipase AMS8 esterification was higher in toluene as compared to L208A. Hence, the results showed that Leu-208 of AMS8 lipase plays an important role in steering a broad range of substrates into its active site region by regulating the flexibility of this region. Leu-208 is therefore predicted to be crucial for its role in interfacial activation and catalysis in toluene.

## 1. Introduction

Increasing interest is being shown in enzymes from organisms living on extreme ecosystems, which resemble the conditions typically found in industrial processes [1]. Amongst these microorganisms is cold-active *Pseudomonas* sp. that produces lipases for biotechnological application because they are versatile enzymes that can catalyze the hydrolysis and synthesis of a wide range of industrially important substrates with broad specificity at low temperature. Due to its low activation energy and structure flexibility properties, their stability in organic solvents has received great attention [2]. One of the efficient strategies to improve the organic solvent stability of a lipase is by increasing the surface hydrophobicity of the enzyme through rational design and directed evolution [3]. However, there is still a lack of a proper theoretical “measure” of hydrophobic interactions with other “intrinsic” interactions which are not easily determined by mutagenesis as the local adjustments may not alter global properties of the protein [4,5]. The engineered enzymes will have limited activity and stability in the presence of organic solvents as they evolved through millions of years in natural aqueous environments. Thus, structural conformations in aqueous environments can be maintained only when the balance between electrostatic and hydrophobic interactions is met [6]. The central role of electrostatic forces are to ensure charges are well distributed for protein recognition as binding itself can induce ionization changes (electrostatic effect) [7]. The effects of single amino acid substitutions on the electrostatic component of the binding free energy would probably allow the substituted residue to experience flexible charge approach which could benefit these enzymes in different pH and salt concentrations [8]. However, the locations of these substitutions vary on a great extent depending on the lipases under examination. A single-residue substitution approach may addresses the fundamental protein engineering questions whether or not it contains potential in improving enzyme activity in organic solvent which eventually can guide researchers into future directed enzyme evolution experiments.

Trade-offs between stability and activity are the general mechanisms of cold-adapted enzymes which provides the reference point for structural understanding. In these enzymes, active sites appear to be the most flexible and heat-labile region than the whole protein. This marks active site as a “hot spots” of mutation for modifying activity [9]. However, the perspective of enzyme thermolability with the company of organic solvent is generally understated as different types of organic solvent may feature specific characteristics on stability and flexibility. To date, there is no direct experimental evidence of an increased flexibility in psychrophilic enzyme although the decreased in stability of cold active enzyme has been well documented [10].

In polar organic solvents, it is assumed that the interaction between the enzyme surface and surface-bound water is disturbed by stripping off the enzyme’s essential water layer. Polar solvents also can interfere with enzymatic activity directly in the active site, while hydrophobic, water-immiscible organic solvents are less detrimental and have even been reported to be consistent with proper enzyme folding and activity [11]. According to general rules for designing mutations in organic media, non-conservative residues that are not involved in the fatal structural elements (i.e., α-helix, β-sheet, and β-turn) or in any critical intra-molecular interactions (salt bridges or hydrogen bonds), while being far away from the active site are commonly used as a target for mutagenesis. The residues located at the said regions are critically susceptible to major structural changes. However, the effects of cold-adaptation, stability and flexibility are likely to vary across different enzyme families. For example, substitution of bulky residues with discrete amino acid in the active site can thermodynamically favour protein-ligand interactions at low temperatures by reducing its K_M_ [1]. Recently, cold-adapted esterases (EstK) which harbored the conserved dipeptide sequence Asp-Tyr located at catalytic His-307 loop was also reported to be responsible in maintaining active site conformation as a result to the establishment of hydrogen bond between D308 and W208. Meanwhile, EstK mutant W208Y situated at the wall of active site exhibited high catalytic efficiency and catalytic site thermal stability as compared to wild-type (WT) [12]. By far, the substitution, insertions and deletions of bulky residues could lead to a more accessible or flexible active site in cold-adapted enzymes. This effect was frequently associated with evolutionary changes where a balance between protein stability and conformational flexibility are mandate for proper function at low temperatures [1].

Although it may be easy to follow rational rules as a standard for cold-active lipase engineering, it is somewhat interesting to observe the diversity in the outcome of amino acid alterations at these critical site points, especially the active sites. Therefore, this paper sought to determine the structure and catalytic changes that follows a single substitution of non-conserved amino acid targeting a nucleophilic elbow region of cold-active lipase AMS8. Both WT and mutant lipases were compared structurally and tested for the potential of low-molecular weight ester synthesis at varying temperature.

## 2. Results and Discussion

### 2.1. Elucidating the Effects of L208A Substitution via Molecular Dynamic Simulation and Protein-Substrate Docking

**Figure 1:**
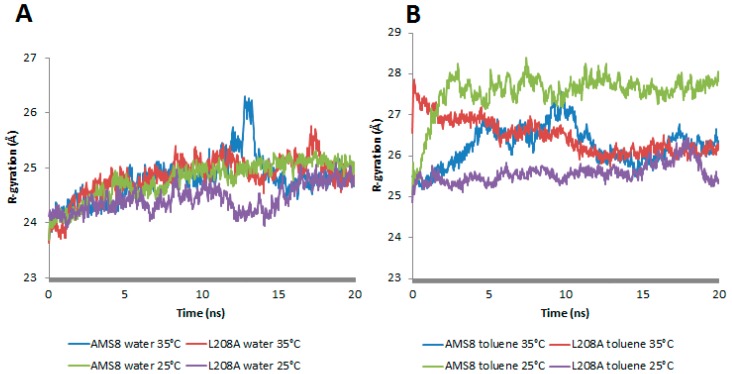
The radius of gyration, Rg (measure of the mass-weighted spatial distribution of the atoms in a peptide molecule/protein and a rough measure for its compactness) in WT AMS8 and mutant L208A in water—(**A**) and toluene—(**B**) (at 25 °C and 35 °C) respectively.

**Figure 2:**
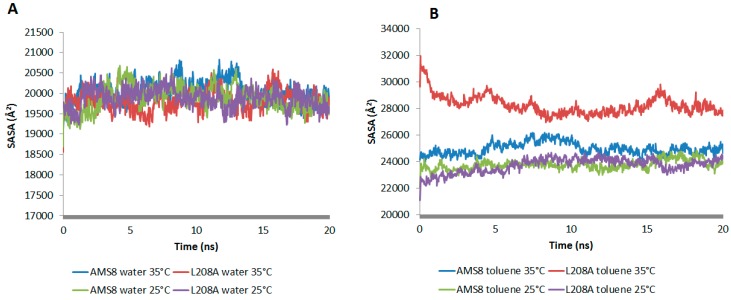
Solvent accessible surface area (SASA) analysis of WT AMS8 and mutant L208A lipases in water (**A**) and toluene (**B**) at 25 °C and 35 °C respectively. Larger SASA indicates highly exposed amino acids to organic solvent.

In-silico analysis of mutant L208A was studied on its molecular basis of stability in the presence of a non-polar solvent, toluene, and at different temperatures. Previously, AMS8 lipase was observed to permit “interfacial activation” in the presence of non-polar solvents. In the presence of toluene, the geometrical properties of WT AMS8 lipase showed a prominent change in terms of the increase in radius of gyration (Rg) and total accessible surface (Figure 1B and Figure 2B). Contrary to water simulation, the WT lipase was more rigid in movement and did not exhibit any major structural changes when the temperature was increased (Figure 1A and Figure 2B).

In general, mutant L208A radius of gyration was stable and sustained at 24.5 Å throughout water-simulation at both 25 °C and 35 °C. As in the presence of toluene, rapid movements of the water molecules that surround the enzyme promote irregularities in its compactness after just 1.5 ns at 25 and 35 °C. It was noteworthy that at 25 °C, L208A lipase exhibited a gradual increase in Rg starting from 25.05 Å to reach the highest value of 26.2 Å while at 35 °C, L208A radius of gyration had a sharp increase from 26.5 Å to 27.8 Å up to 1.6 ns, but finally moved back to its normal form at 25 Å after much extended simulation as shown in Figure 1B. Hence, the mutant L208A lipase could prevent its structure from losing its native compactness in the presence of toluene at moderately high temperature (35 °C). It was believed that the improvement of compactness on L208A lipase at 35 °C has something to do with the active movement of toluene that surrounds the enzyme due to its lower volatility (higher boiling point) as compared to water molecules. Following this result, L208A lipase was more likely to favour a slight increase in temperature as shown by its structure compactness in toluene.

The accessible surface areas of atoms are correlated with their hydrophobicity and folding process. The folding process is usually accompanied by a significant decrease in SASA value. In water simulation, both WT AMS8 and L208A lipase showed to have similar SASA pattern at 25 °C and 35 °C, which is indicative of having no difference in the folding process (Figure 2A). However since toluene was added into the simulation, the average value of SASA increased by 1 to 1.5 times (Figure 2B). Even though both WT and L208A lipases exhibited an increase in SASA when simulated at 35 °C, the latter one was shown to have undergone maximum local rearrangements that modify the energy interactions with the solvent which could influence the state of protein folding itself.

**Figure 3:**
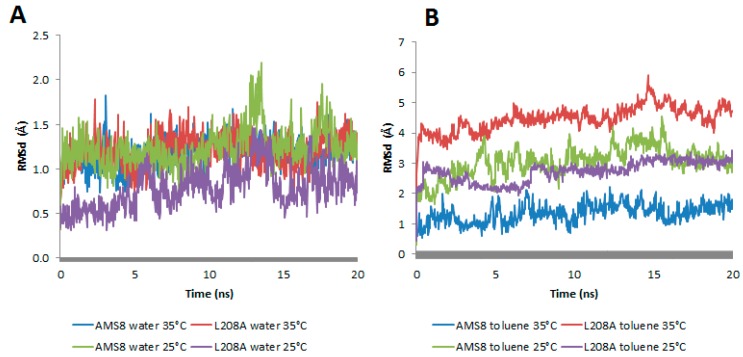
The root-mean-square (RMSd) distance between the Cα atoms in the simulations and the lid 2 (148–167) structure of AMS8 and its mutant L208A in water—(**A**) and toluene—(**B**) at 25 °C and 35 °C respectively.

There are higher chances of surface area contact between lipases in toluene as compared to water. Although the area function is not specific to the structures of proteins, the derivation was motivated by the need for a computationally feasible simulation of the hydrophobic effect in proteins [13]. Therefore, lid 2, a region known for its hydrophobicity interactions, was targeted and compared for its root-mean-square deviations (RMSd) at both conditions. In general, both WT and mutant L208A lipases displayed an average distance of 1–1.5 Å in water simulation at 25 °C and 35 °C (Figure 3A). This analysis revealed that water stabilizes the lid at both temperatures. A change in lid stability of mutant L208A lipase could be found prior to temperature changes in toluene as shown in Figure 3B. The difference in thermal condition happens to change mutant L208A lid stability at 35 °C following the escalating RMSd value. As a result of this, mutant L208A was predicted to have experienced a disturbance in lid stability and the possibility of undergoing thermal denaturation, while at lower temperature (25 °C), lid 2 of L208A lipase was shown to stabilize and reach equilibrium with the RMSd value of the wild-type strain. WT AMS8 lipase exhibited a further decline in RMSd values when simulated at 35 °C as shown in Figure 3B and this effect could be associated with the thermolability as high temperature that abolishes the stability of cold-active lipase. Based on molecular dynamics, a large difference of RMSd between WT and L208A lipases had raised concerns about the potential impact of a single mutation at the nucleophilic elbow region on its ability to alter the lid stability at different temperature settings.

**Figure 4:**
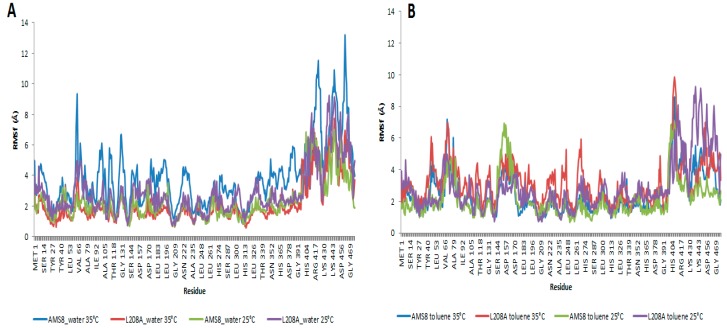
The root-mean-square (RMSf) distance between the Cα atoms in the simulations and the protein structure of AMS8 and its mutant L208A in water—(**A**) and toluene—(**B**) at 25 °C and 35 °C respectively.

Root-mean-square fluctuations (RMSf) of the Cα atoms from residues 1–476 were calculated for the two simulation parameters. In water, WT AMS8 lipase showed a larger fluctuation starting at residues Ser-60 to Gly-65 when simulated at 35 °C compared to a similar simulation running at 25 °C. The increased motion in the simulation was mainly due to the polar properties of the affected amino acids which have been stimulated by the vibration of water molecules promoted by the increase in temperature. These regions did not embody any essential core structure such as lid region that was homology modelled and verified for its position at residues Ala-51 to Leu-57 and Glu-148 to Gly-167 which was similar to that of *Pseudomonas* sp. MIS38, PML [14]. The fluctuations would not probably change any conformation of the protein, but both N- and C-terminal domains were disturbed by the large amplitude motions caused by the temperature change. As for L208A lipase, similar residues were not perturbed by the change of temperature as observed in Figure 4A. This caused the protein to remain stable in water. In toluene, the increased motions mainly come from three sites; the first site was lid 1 (residue 51–57), second site was Ser-60 to Gly-65 (as observed in water simulation) and the third one is lid 2 (Glu-148 to Gly-167) as shown in Figure 4B. Key difference could be observed at the first and third sites where toluene which has been earlier found to promote lid 2 activation was also found to concurrently stimulate lid 1 opening. Apparently it hits the opening of lid 1 at temperature 35 °C as the change affects coordination of related amino acids. It was discovered at 35 °C that both WT AMS8 and L208A lipases could achieve double-lid movements in unison. For that, it is predicted that a complete interfacial activation occurred through the opening of shorter-range lid 1 and longer-range lid 2 at higher temperature can allow the access of a broader range of substrates which makes the enzyme readily receptive for various biochemical industry applications, especially those that involve toluene. After all, the water-oil interface concept may not seem to be the major requirement in enabling the movement of the lids and providing access to the catalytic pocket of the WT AMS8 lipase [15].

Following the current interpretation, the superimposed of L208A lipase at 0 and 20 ns was structurally compared and from here, the three fluctuated sites or sequences were identified as shown in Figure S1A. The three aforementioned sites were observed as the most flexible region as shown in the protein structure clustal alignment (Figure S1B). Most importantly, all three catalytic residues Ser-207 (positions 207–214), Asp-255 (254–255) and His-313 (314–315) positions have completely changed and the enzyme experienced losing the native structure with the substitution of Ala-208. Thus, it was also predicted that mutant Ala-208 could hugely affects the catalytic rate via modification of substrate binding sites.

**Figure 5:**
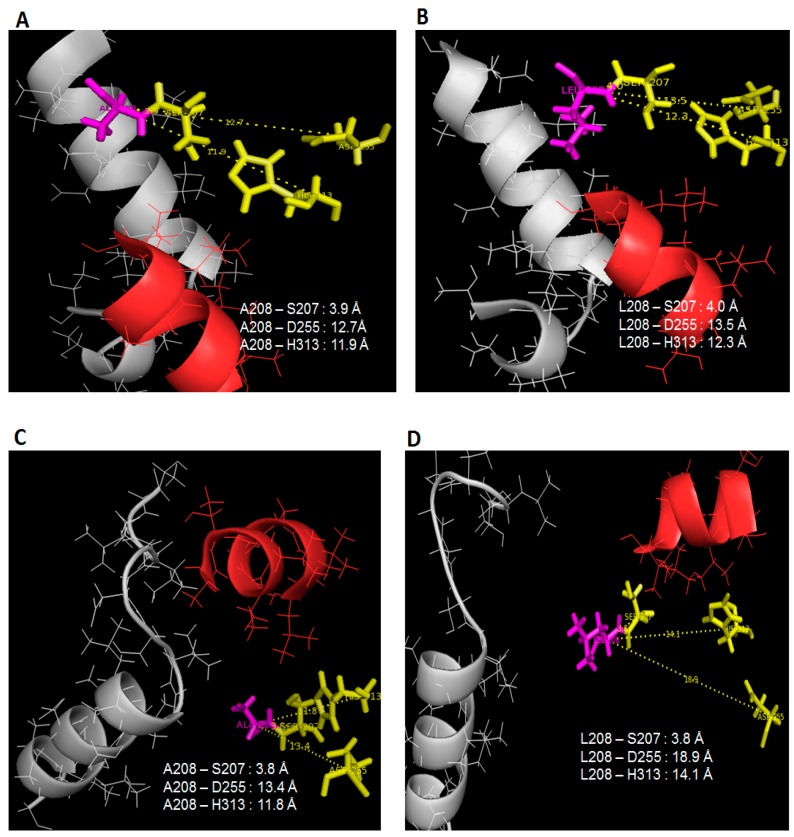
Distance of L208/A208 with catalytic triad S207, D255, H313 in 25 °C simulation. Image (**A**,**C**) referred to L208A simulated in water and toluene while (**B**,**D**) pointed to AMS8 lipase simulated in water and toluene. Lid 1 (51–57) was represented in red helix, lid 2 (148–167) was in grey helix, L208/A208 residue in purple stick and catalytic triads (Ser-207, Asp-255, His-313) were identified as yellow sticks.

**Figure 6:**
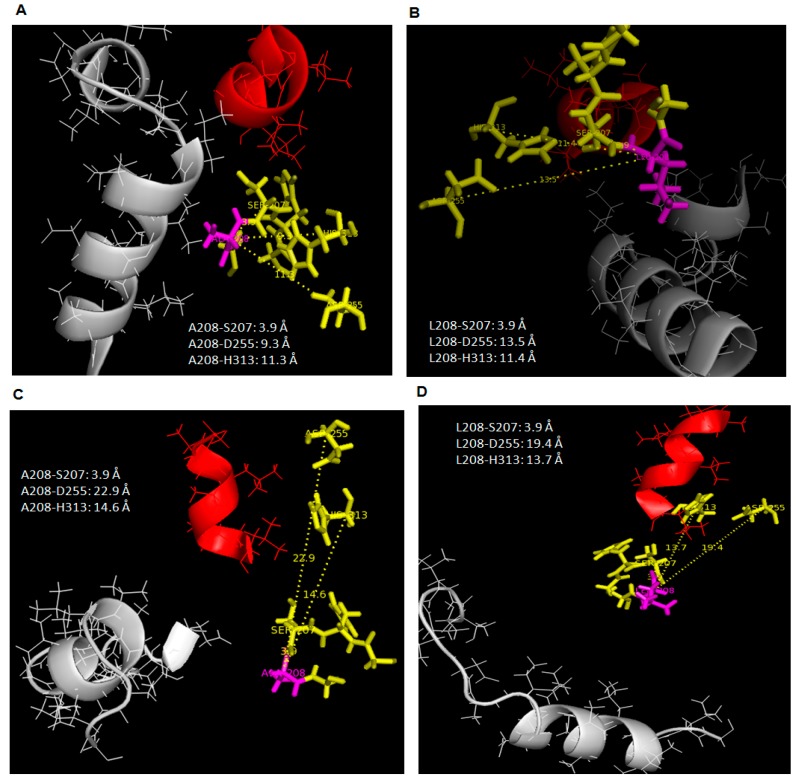
Distance of L208/A208 with catalytic triad S207, D255 and H313 in 35 °C simulation. Image (**A**,**C**) referred to L208A simulated in water and toluene while (**B**,**D**) pointed to AMS8 lipase simulated in water and toluene. Lid 1 (51–57) was represented in red helix, lid 2 (148–167) was in grey helix, L208/A208 residue in purple stick and catalytic triads (Ser-207, Asp-255, His-313) were identified as yellow sticks.

The conformational dynamics of wild type AMS8 and mutant L208A lipases showed that minimal reorganization and flexibility of lid regions was needed when the temperature was set 10 °C higher. Furthermore, a slight increase in temperature made the active sites of L208A lipase less elastic in the presence of toluene and water (supported by MD). As for wild type AMS8 lipase in water, no difference in terms of folding state and rigidity was observed when the temperature was adjusted, indicating that AMS8 lipase remained stable within the given temperature range. Many studies have inferred that the conformational flexibility of a cold-active protein is a direct consequence of conformational stabilization, where protein stability is associated with a decrease in conformation flexibility and a reduction in enzyme activity [16], but in this scenario, it is rare to see such improvements in protein stability and flexibility (only at lid 1 area) almost at the same time which coincidentally was instigated by temperature changes. This finding relates to the restructuring of active sites position which alter the specific-substrate binding abilities. Following the lack of fluctuations surrounding the lid 2 region in 25 °C simulations of L208A lipase, it was found that the space that separates the nucleophilic serine from the rest of the catalytic residues has been reduced significantly, as shown in Figure 5A,C. Once Leu-208 was changed to Ala-208, the distances between Leu-208 to Asp-255 and His-313 were reduced by 0.8 Å and 0.4 Å, respectively, in water (see Figure 5A,B), while in toluene, the distance differences between these triads were much greater because lid 2 was opened in WT AMS8 lipase but closed in L208A lipase as a result of Ala-208 substitution which demonstrates a distance difference of 5.5 Å and 2.3 Å to Asp-255 and His-313, respectively (Figure 6C,D). Even though the geometry of the catalytic sites (includes catalytic triad and the nucleophilic elbow motifs) is highly conserved in mutant L208A, the size and shape of the binding site differ considerably. Wild-type AMS8 lipase binds fatty acids by shoving the substrate into a long stretchy funnel-shaped binding site with contribution by hydrogen-bonds formed between Ser-238 and Gly-210 (Figure S2A). However, in the absence of hydrogen bonding between Ser-238 and Gly-210 in the L208A mutant, the binding-site of this fatty acid was blocked due to the collapse of this tunnel (Figure S2B). Most lipases are classified according to the binding of fatty acid moieties (from esters) into three classes, where lipases could form a hydrophobic, crevice-like binding site located near the protein surface such as the lipases from *Rhizomucor* and *Rhizopus*, or lipases with a funnel-like binding site, such as the lipases from *Candida antarctica*, and *Pseudomonas* or finally lipases with a tunnel-like scissile fatty acid binding site such as the lipases from *Candida rugosa* and *Geotrichum candidum* [17]. The molecular basis of the fatty acid binding site and the role of Leu-208 in chain length specificity will be discussed later by observing the ethyl hexanoate docking analysis and kinetic profiles of various *p*-nitrophenol substrates, with and without toluene (K_M_ and K_cat_).

The temperature dependence of catalytic activity in lipase has been widely reported by researchers, with most reporting that site-directed mutagenesis targeting catalytic residues would disfavour nothing but the catalytic efficiencies. The advantage of this technique would be to improve the structure stabilization and a shift in temperature dependence [18,19]. Cold-active lipases were originally adapted to thrive and produce well in cold environments but due to the limitations cause by the thermolability properties, this hindered the enzyme from achieving the proper balance between stability, activity and conformational flexibility. It is now clear that AMS8 lipase is exceptionally stable and active in the temperature range between 20–25 °C as indicated by previous temperature characterization profiles [14], but the enzyme shows that either temperature or solvent itself could initiate a significant effect on the structure stability or flexibility until a single point mutation was introduced [15,20].

As a consequence of having a mutated residue Ala-208, the catalytic residues of this lipase became slightly closer in distance when simulated in water and even more closer in solvent. This happened within the optimal temperature range required by AMS8 lipase for catalytic function (20–30 °C). As the temperature increased, the changes in the distance of catalytic triads could be affected by the gradual loss of proper folding and a decline in structure flexibility of this cold-active lipase. Flexibility is crucial for activity at low temperature and such alterations or substitutions made on the active site and its neighbor residues could potentially lead to substrate binding obscurity and inefficient catalysis at low temperatures. Thus it is imperative to keep the motion unrestricted [5].

At 35 °C, all trajectories at 20 ns showed that the distance between Ala-208 to catalytic Asp-255 and His-313 had become closer than it has been (Leu-208) initially at 25 °C when simulated in water. This effect was similar to WT AMS8 lipase simulated in water (Figure 6A,B). As a result, the catalytic binding sites will become more rigid at high temperature. Hence, this results showed an agreement with the stability (RMSd) gained by both wild-type and mutated lipases when simulated at higher temperature in the presence of water. However, the catalytic residues (Asp-255 and His-313) of both AMS8 and L208A lipases were found to drift further away from the position reported in toluene at 25 °C (Figure 6C,D).

### 2.2. Electrostatic Potential and Hydrophobic Interaction Distribution of L208A and WT in Toluene

Differences in electrostatic potentials in and around the active site of psychrophilic enzymes appear to be a crucial indicator for activity at low temperatures. Electrostatic surface potentials generated by charged and polar groups are an essential component of the catalytic mechanism at various stages: as the potential extends out into the medium, a substrate can be oriented and attracted before any contact between enzyme and substrate occurs [21]. As for hydrophobic effects, they are an important factor that influences protein folding and solubility. The solubility of non-polar compounds in water is small due to the large decrease in entropy associated with the formation of clathrate hydrate caging surrounded the nonpolar residues [22,23]. It is through the increase in entropy or collapse of the cage-like structure that will bring to the formation of E-S complex at the active site function as a driving force in substrate binding. This effect is called hydrophobic interactions and the higher the contribution of hydrophobic effects in a binding process, the higher will the organic solvent effect turn out to be as reflected by the changes of K_cat_ [24]. Thus, the changes in hydrophobic and electrostatic energy by toluene would be reflected based on the K_M_ and K_cat_ of both L208A and WT lipases. At 25 °C, it was found that electrostatic energy (kJ/mol) has a non-linear correlation with hydrophobicity (via fraction of matching hydrophobic surface) as indicated in Table S1. Both L208A and WT lipases were shown to have a lower hydrophobic surface match when reacted in toluene as compared to water at 25 °C. This means the hydrophobicity interaction between the lipase and the substrate, ethyl hexanoate, is not improved by mutating Leu-208 to alanine, partly due to the absence of a hydrophobic side chain. The turnover number, K_cat_ and substrate affinity, K_M_ of L208A lipase that utilizes *p*-nitrophenol caprylate (C-8) declined progressively in the presence of toluene, but increased in water. The decline of both K_M_ and K_cat_ of mutant L208A could be attributed to the preservation and growing numbers of hydrophilic surface which greatly opposing the polarity of toluene. With high electrostatic energy, the hydrogen-bond formed between enzyme and substrate increases. Thus, this condition promotes the distribution of hydrophilic contacts developed between the substrate and enzyme in the presence of toluene.

### 2.3. Substrate/Product-Enzyme Binding

Part of the rate limiting step in lipase activity is the formation of a tetrahedral intermediate stimulated by the nucleophilic attack of the catalytic serine [25]. A tetrahedral intermediate (or some called it oxyanion hole) forms in the lipase to drive the ester hydrolysis catalysis. It is the formation or collapse of the TI that determines the substrate selectivity [26]. The binding pockets of lipase provide a prearranged environment to specifically stabilize this intermediate by hydrogen-bonding. Therefore, a predictive model for WT AMS8 and mutant L208A lipase that rigidly docks its substrate has to take into account whether this archetype reflects the interaction of enzyme in stabilizing the transition state which causes the anionic carbonyl oxygen to move deeper into the active site. Esterification may take place, once a tetrahedral enzyme-substrate (ES) intermediate is stabilized.

**Figure 7:**
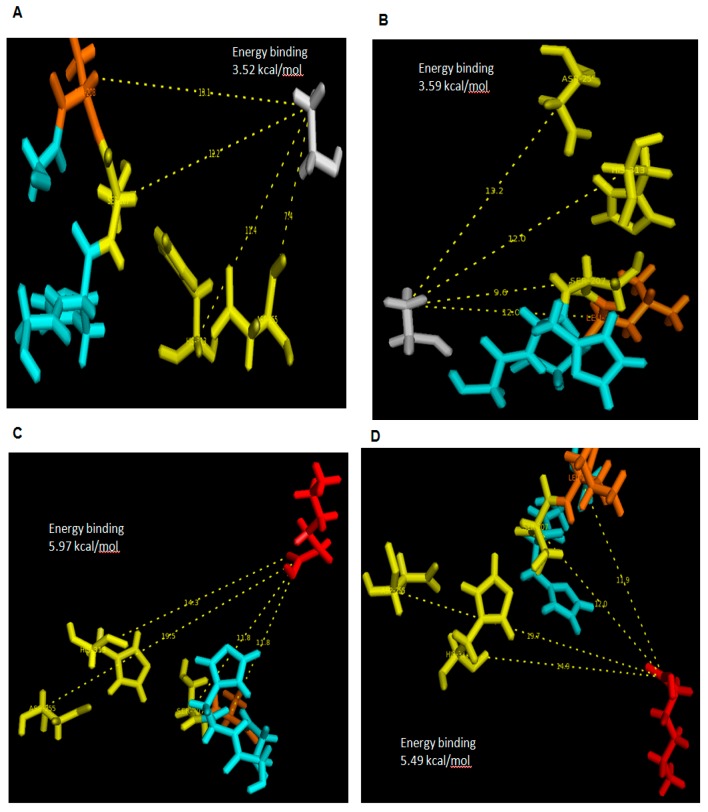
Docking with substrates ethanol and hexanoic acid showing distance with catalytic residues, Ser-207, Asp-255 and His-313. (**A**,**C**) referred to L208A docked with ethanol (grey) and hexanoic acid (red) while (**B**,**D**) showed WT AMS8 lipase docked with ethanol and hexanoic acid. Ethyl hexanoate was identified in stick colored in red, all catalytic residues in yellow, mutated or non-mutated Leu-208 in orange (with re-orientated side chain) and surrounding residues of GXSXG motifs (Gly-205, His-206, Gly-209) were identified in turquoise.

**Figure 8:**
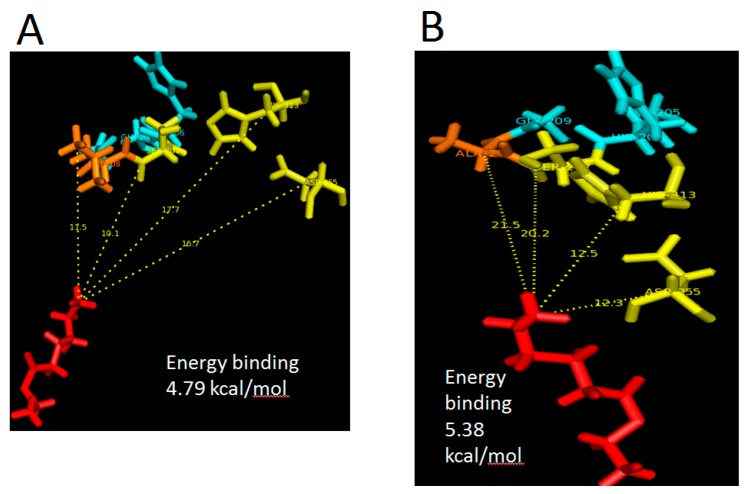
Docking of wild-type AMS8 (**A**) and mutant L208A (**B**) with ethyl hexanoate (C_8_H_16_O_2_). Asp-255 and His-313 were seen moving closer to substrate and the nucleophilic elbow (**B**). Ethyl hexanoate was identified in stick colored in red, all catalytic residues in yellow, mutated or non-mutated Leu-208 in orange (with re-orientated side chain) and surrounding residues of GXSXG motifs (Gly-205, His-206, Gly-209) were identified in turquoise.

In this conventional docking, alcohol and acid substrates were covalently docked into WT and mutant L208A lipases separately at designated active site regions. After docking was completed, the binding energy involved between each substrate and lipase was determined and the value signifies the energy forces required for the formation of intermediates (enzyme + substrate). Based on the docking analyses, it was found that ethanol has a binding energy with WT AMS8 and L208A lipases of 15.02 and 14.73 kJ/mol, respectively. As for hexanoic acid, WT AMS8 and L208A were reported to have binding energies of 22.97 and 24.98 kJ/mol (Figure 7), but when the product in the form of ethyl hexanoate were covalently docked into WT AMS8 and mutant L208A, approximate energies of 20.04 and 22.5 kJ/mol were needed for the active site binding and achieving the transition state (enzyme + product) (Figure 8A,B). According to Koshland, the active sites of some enzymes assume a shape that is complementary to that of the transition state only after the substrate is bound. Thus, a slight increase in energy binding between mutant L208A catalytic residues and ethyl hexanoate can be associated with the lack in flexibility and steric constraints of the surrounding amino acids that form the active site [27].

The relative binding-free energy can change prior to its binding landscape. In this case, both proteins are assumed to be rigid while the substrates are treated as a flexible molecule. In these docking analyses, it was clearly shown that Leu-208 was responsible for the binding efficiency. In the transition state, Ala-208 was placed further away from ethyl hexanoate as compared to Leu-208. The presence of a side chain in Leu-208 allows the said residue and nucleophilic serine to be placed 10 Å closer to the substrate. Meanwhile, in L208A, Asp-255 and His-313 exhibited a 5.2 and 4.4 Å closer distance with a similar substrate. Due to this reason, mutant L208A has been predicted to possess better substrate recognition due to the clustering of active site residues. However, it is also thinkable that the production or conversion rate of substrate to product will become less efficient as there will be delay for the nucleophilic serine (Ser-207) to attack the substrate due to its further distance. Mutation at substrate-binding pocket (I100F) of *Yarrowia lipolytica* Lip2 lipase showed that the distance of nucleophile attack between Oγ of S162 and the carbon atom of carbonyl group in the monoester substrate, pNPC12 (I100F-pNPC12 complex) was reported to be 3.7 Å. As for WT-pNPC12 complex, a shorter distance of 3.0 Å was recorded. Similarly, the distance affects the molecular collision during reaction due to the difference in amino acid’s side-chain, decreasing the activity of I100F towards pNPC12 [28]. However, Kumar et al. [5] indicated that G155D substitution of lipase A from *Bacillus subtilis* located at the active site assisted in a faster release of product due to the electrostatic interactions with neighboring amino acids. Therefore depending on the location of the mutation site, the conformational changes of the active site can contribute towards a variety of effects.

The strong binding energy between L208A and ethyl hexanoate also mean that this mutant lipase could spare more energy to break down the enzyme-product (EP) intermediate as a means to release the product. In this study, it is predicted that mutant L208A can cause a longer time in the formation and collapse of the intermediates (transition state) as compared to WT AMS8 lipase. Most frequently, changes that are related to active sites leads to a more accessible and/or a more flexible active site if the substitution, insertions and deletions of amino acid were made with a preference toward bulky residues [1]. This study however, annuls such a concept in promoting active site flexibility by substituting Leu-208 with lanine, a residue adjacently located by catalytic Ser-207.

It is important that only residues which come in close contact with the substrate could tell the difference whether this substrate could form any interactions with the protein, particularly the hydrophobic ones. In general, comparisons of the best scoring docked poses for primary substrates ethanol and hexanoic acid as well as final product ester, ethyl hexanoate are shown in Table 1. Only hexanoic acid shared a similar interaction with both proteins and the substrate apparently targeting Tyr-29, His-30, Leu-32, Ser-60, Thr-61, Ser-63, Gln-64, Gly-65, Trp-72, Ser-76, Glu-77, Arg-120 and Arg-141 which mostly located at N-terminal and these interactions were found at the surface of the two proteins. This series of residues especially positioned between 60 and 70 are highly flexible and was always moving according to B-factor analyses. These areas are rich in polar residues which could be important in forming hydrogen bond interactions between acyl group of hexanoic acid with the hydroxyl group of polar residues. In the presence of ethanol, only L208A has its catalytic site, Asp-255 interacting with it followed by subsequent residues located between Asp-255 and His-313. WT AMS8 lipase has the most contact with the ester ethyl hexanoate, particularly at the lid-2 region (Leu-166 and Gly-167) where none of this interaction occurred in mutant L208A. Thus, the substitution of residue 208 hugely affects the number of productive poses between mutant L208A and ethyl hexanoate.

In order to verify the influence of organic solvent as part of docking module, the combination of lipase/toluene/substrate system was applied by using the saved trajectory of lipase simulated in toluene to execute docking with substrates. These results would reveal the difference of substrate binding in the presence of toluene as the lipase used mimicked the one which reacted in the system. Based on the docking analysis, WT AMS8 lipase shared few contacting residues with its two substrates, and most of them were targeting residues buried inside the core of the N-domain as shown in Table 2. This result showed that the interaction established between the substrates and buried residues of AMS8 lipase was driven by large opening left by lid 2 after simulations in toluene (Figure S2A). Interestingly, mutant L208A surface residues located on lid 1 and lid 2 were identified as the major point of interactions that connect substrates with mutant lipase. The outcome was logical because mutant L208A provided no admission or path that could lead the two substrates straight into the catalytic area (Figure S2B). Since lid 2 did not give ways for substrate to get inside, it was impossible to observe the binding of substrate with any residue within the catalytic surroundings. As for the end-product, ethyl hexanoate was not subjected to binding with the catalytic area in WT AMS8 lipase as it targets the residues located at N-terminal sites (situated between residues 30–78). The complex lipase docking with substrate in the presence of organic solvent has been applied in a few studies, but mostly with the objective of viewing interactions and enantioselectivity prospects in controlled systems such as microemulsions or immobilization [29,30].

### 2.4. Kinetic Profiles of L208A

To demonstrate the presence of catalytic promiscuity or substrate specificity of L208A, some relevant substrates defined by the length of carbon chain of *p*-nitrophenol esters were considered for analysis. Based on the preliminary screenings, AMS8 lipase has the highest specific activity with substrates: natural oil and pNP caprylate in the presence of 25–30% (*v*/*v*) toluene, whereas L208A lipase displayed poor specific activity in natural oil with and without the presence of toluene. L208A lipase was found to exhibit a moderate activity in pNP-caprylate without toluene and showed highest activity with a medium-chain ester which was pNP-laurate (Table 3). Because of these variations in substrate preference, both lipases were subjected to Michaelis-Menten kinetics to measure substrate affinity (K_M_), turnover number (K_cat_) and K_cat_/K_M_. According to the kinetic analyses, L208A demonstrated the lowest K_M_ and highest K_cat_/K_M_ for the C-8 substrate (Table 4). However, both V_Max_ and K_cat_ was found to be low, which explained the obstacle in getting a high enzymatic rate. Hence, the kinetic outcomes of mutant L208A follows in agreement with the previous protein-substrate docking analyses in associating the strength of substrate binding with energy (kJ/mol) or time (second) spent to liberate the product as a final output. In toluene, an increase of K_M_ value in both WT AMS8 and mutant L208A were displayed according to the substrate’s numbers of carbon-chain as it favors the smallest chain. In this case, mutant L208A has failed to promote better hydrolysis reaction in the presence of long-chain pNP substrate and toluene. For WT AMS8 lipase, the changes in K_M_ had significantly lower the V_max_ but did not result in any significant improvements to the K_cat_ and K_cat_/K_M_. The K_cat_/K_M_ in AMS8 lipase was meagerly raised in toluene in the presence of all three pNP substrates. In contrast, mutant L208A showed a high value of K_cat_/K_M_ with toluene only in the presence of caprylate ester.

Based on this kinetic analysis, it is interesting to learn that mutant L208A is able to hydrolyze the short pNP substrate at high K_cat_/K_M_ (and low K_M_) although facing disadvantages in the formation of a rigid active site. The idea of having a rigid active site capable of supporting higher enzyme activity in a cold-active lipase is quite contrary. Supposedly mutations that leave more space lead to a better improvement of enzyme activity [12]. As for L208A mutation, the active sites and lid 2 regions had been reorganized or reshaped to a level of extreme rigidity which has been addressed in both molecular dynamic and docking studies. However, the mutant was still capable of making the smallest (C-8) chain substrate bind and hydrolyzed it with the highest efficiency. Both long and medium-chain substrates are thought to have difficulties in accessing the active sites due to the closing of the lid and shrinking space inside the catalytic pocket.

### 2.5. Esterification of Ethanol and Hexanoic Acid

**Figure 9:**
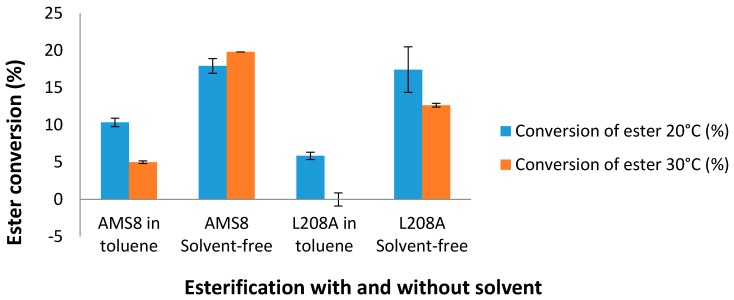
Esterification conversion rate of mutant L208A with and without toluene using 5 mg of protein per reaction at 20 °C and 30 °C.

In the manufacturing industry, hexanoic acid is commercially used for the production of esters used in perfumery and in the manufacture of dyes. Therefore, L208A mutant was subjected for low-molecular weight esterification with and without the presence of toluene. Esterification of L208A lipase with the substrates ethanol and hexanoic acid at 20 °C showed that the rate of ester conversion was very much alike with AMS8 lipase in a solvent-free system as shown in Figure 9. AMS8 lipase was found to be active over a broad temperature range of 20–30 °C [31]. As the temperature was increased to 30 °C, the ester conversion was found to be improved by 10.5% in the solvent-free WT AMS8 lipase esterification. However, the ester conversion was reduced by 27.4% compared to the one synthesized at 20 °C in mutant L208A lipase. In toluene, WT AMS8 lipase showed a gradual decay of ester production while L208A was found not to produce esters at this temperature. Higher ester conversion in WT AMS8 lipase means that having highly flexible active site is important to facilitate rapid production of ethyl hexanoate. Thus, the smaller active site and rigidity of lid 2 from mutant L208A may not ease the effect of a rate limiting step on the reaction rate [22]. Currently, both lipases are found not to be effective ester producers in toluene.

The replacement of Leu-208 by alanine caused a negative effect on lipase activity in the presence of toluene following the falling trend of ethyl hexanoate esterification. This outcome was anticipated as kinetic studies also showed that toluene contributes to a lower substrate binding affinity. Mutant L208A lipase activity however was found to increase by means of K_M_ and K_cat_ of pNP-caprylate hydrolysis at 25 °C and preserved ethyl hexanoate esterification at 20 °C showing that it works well in the 20–25 °C temperature range. An increase in temperature (30 °C) could cause rapid atomic movements of solvent (in toluene) and a loss of important interactions due to the shifting. A simple molecular dynamics of mutant L208A in the presence of ethyl hexanoate at 20 and 30 °C clearly exhibits a change in carbon backbone stability as shown by the RMSd result in Figure S3A. At 30 °C, L208A experienced a major structural change after 7.5 nanoseconds of simulation preventing the mutant from having a structure amenable to ligand binding. A contrast RMSd at 20 °C, showed a flat graph with values from 2–4 Å could be an indication of an optimized structure for ligand-protein binding. The same results could be observed in the fluctuation ratio of residues in Figure S3B where all of the structure consistency such as ligand binding, energy and interaction was highly dependent on it. Adding to the findings of L208A, the mutant also demonstrates a large radius of gyration or less compactness in structure at 30 °C (Figure S4A). In actual fact, the compactness is a definition of a ratio of the accessible surface area of a protein to the surface area of the ideal sphere of the same volume. This may suggest that L208A has an indiscriminate region or broader sites in allowing ethyl hexanoate to bind at 30 °C. According to Lobanov et al. [32] a compact packing of amino acid residues is known to affect both the stability and folding rate of proteins. In addition, α-proteins fold more rapidly and are less compact as compared with the β- and (α + β)-proteins, while α/β proteins have the lowest folding rate and are most compact. In this analysis, L208A demonstrates a consequent loss of α (3%) and β (1%) structure at 30 °C which had contributed towards loose packing and possibly affect its folding (Figure S4B). Since α/β-proteins would display the lowest radius of gyration and tightest packing, a decline in α/β structures should have a major consequence in the compactness of protein structure.

## 3. Material and Methods

### 3.1. Purification of Recombinant AMS8 Lipase

#### 3.1.1. Preparation of Enzyme from Inclusion Bodies

The *E. coli* strain BL21 (DE3) cells harboring the recombinant plasmids was grown in 300 mL of Luria-Bertani medium containing 50 µg/mL ampicillin at 37 °C and 200 rpm agitation until the OD_600_ reached 0.5. The culture was then induced at 20 °C with 0.1 mM IPTG for 12 h. Next, the cells were centrifuged at 10,000× *g* for 10 min. The cell pellets, collected by centrifugation, was resuspended in 30 mL of 50 mM Tris-HCl buffer (pH 8) and disrupted with 10 s pulses for 6 min of ultrasonic disruption, with the container containing the cell resuspension fixed in an ice case. The intact cells were removed by centrifugation at 10,000× *g*, and temperature was also maintained at 4 °C for 10 min. The supernatant was decanted and the cell pellets were later resuspend in the same buffer but contained 5 mM imidazole, 0.5 M NaCl and 6 M urea for protein solubilization. The 30 mL of mixture was constantly shaken for a minimum of 3 h at 150 rpm in 4 °C water-bath. Later, the refolding of the solubilized protein was performed either by 10 fold dilution of the binding buffer or by dialysis overnight [31].

#### 3.1.2. Purification of Recombinant AMS8 Lipase

Crude insoluble AMS lipase was purified by affinity chromatography (Nickel Sepharose^TM^ excel, GE Healthcare Bio-Sciences, Uppsala, Sweden). The binding buffer contained 50 mM Tris-HCl, 0.5 M NaCl and 5 mM Imidazole (pH 8.0) was used for column XK 16/20 equilibration. Washing buffer contained 50 mM Tris-HCl, 0.5 M NaCl, 5mM CaCl_2_ and 50 mM imidazole (pH 8.0) and elution buffer contained 50 mM Tris-HCl, 0.5 M NaCl and 500 mM imidazole (pH 8.0). Elution fractions containing targeted protein was pooled and concentrated through a Macrosep Advance device (PALL Life Sciences, Port Washington, NY, USA) with a protein size cut-off of 30 kiloDalton. The single-step purified L208A and WT lipases were subjected to lipase assay.

### 3.2. Site-Directed Mutagenesis

Mutants of AMS8 lipase was developed by a QuikChange Lightning Site-Directed Mutagenesis site-directed mutagenesis kit (Catalog No. #210519, Agilent Technologies, West Cedar Creek, TX, USA). The rapid PCR cycling parameters of the QuickChange Lightning enzyme blend were used, together with other components, QuickSolution, 10× QuickChange Lightning reaction buffer and dNTP mix up to total reaction of 25 µL. The PCR reactions consists 12.5 µL of 10× QuickChange Lightning reaction buffer, 0.5 µL Quik Solution, 0.5 µL of each 10 nM forward and reverse mutagenic primers, 1 µL dNTP, 1 µL QCL enzyme blend and distilled water. After the amplification, 1 µL of Dpn I restriction enzyme was added directly to each amplification reaction. The reaction mixtures were spin down for 1 min and immediately incubated at 37 °C for 5 min to digest the parental ds-DNA. Selection of cloning hosts was either XL10-Gold ultracompetent cells or TOP10 (Invitrogen, Carlsbad, CA, USA), and they are thawed to 30 min on ice before the PCR products were added in. The Dpn-treated DNA (1.5 µL) from each mutagenesis reaction was transferred to the thawed competent cells. The transformation cells were mixed by gentle swirl and incubated for 30 min on ice. Heat-pulse at 42 °C was performed in water bath for 30 s (XL-Gold) and 1 min (TOP10). If XL10-Gold ultracompetent cells were used, pre-heated NZY broth was used for the incubation at 37 °C, while free ampicillin LB or SOC medium (Invitrogen) was used in transformation cells of TOP10. These tubes were incubated at 37 °C for one hour with shaking at 220–250 rpm. Then, 50 µL of each transformation reactions were plated on agar plates with appropriate antibiotics.

The targeted site L208A from catalytic site was selected for the mutagenesis experiment based on the structure-guided consensus (YASARA) and imutant prediction software to identify the residue that most probably governed the structural stability and catalytic activity of LipAMS8. The resulted mutants will be added to similar vector as the native (pET32b), and followed by transformation into BL21 (DE3) competent cells. The designed primers (5’–3’) for mutagenesis are CAGCGGTCATAGCGCGGGTGGCCTGGC for forward and GCCAGGCCACCCGCGTATGACCG CTG for reverse with the annealing temperature of 70 °C. In order to calculate a stability score for mutant proteins, Site Director Mutator (SDM), a free server for predicting effects of mutations on protein stability and malfunction was applied (http://mordred.bioc.cam.ac.uk/~sdm/sdm.php).

### 3.3. Molecular Dynamics and Docking Analysis

The MD simulations were performed using YASARA Structure software (version 11.3.22, Krieger, Nijmegen, The Netherlands) [20]. For each box filled with solvent, a periodic boundary condition was created. Another 6 molecules of Ca^2+^ and 1 molecule of Zn^2+^ were added in the trajectory box (90° on every axis) to neutralize the electron repulsive forces. The solvent densities were all fixed according to their respective relative densities at 25 °C and 35 °C, in which the surrounding spaces was later filled by a mixture of water and NaCl molecules via simulated annealing minimization of solvent at pH 8.0. In this process, short simulation was performed to adapt in system in which water molecules were deleted. Then the O_2_ from water was turned into ion to enable NaCl to be at 0.9% concentration. As for simulation in toluene, solvent density of 0.8621 g/cm^3^ was set at 25 °C and 0.8527 g/cm^3^ at 35 °C, both with 0 bar of pressure control. Meanwhile, protein simulation in water used 0.997 g/cm^3^ as its density at both temperature at pressure control of 1 bar.

During minimization experiment, YASARA temporarily adds a water shell and optimizes it for use with explicit solvent. The initial structure of predicted AMS8 was energy-minimized for 962 steps of steepest descent followed by 962 steps of conjugate gradient of optimization before the actual simulation began. After minimization, the MD simulations were performed following the achieved density equilibration in a fixed-size simulation box at 90° on every axis. At 298.15 K (25 °C), the simulation systems were performed under the steepest descent parameters, using a time step of 1 fs for intramolecular forces which calculated the intermolecular forces in every 2 simulation sub-steps. The structure was energy-minimized with AMBER03 force field using a cut off 10.486 Å and the Particle Mesh Ewald algorithm to treat long range electrostatic interactions. After the removal of conformational stress by a short steepest descent minimization via 50 steps of MD in solvent, the procedures continued by simulated annealing and converge as soon as the energy improves by less than 0.05 kJ/mol per atom during 200 steps. The changes of protein structure was examined using 800 saved steps for each simulation with (25 picoseconds times 40 steps) represents 1 nanosecond. The simulation was allowed to run until 20 ns. The AMS8 lipase homology model has two lids and was in a close conformation [15].

The AutoDock method follows the Lamarckian Genetic Algorithm and Empirical Binding Free Energy Function. Force field Amber03 (YASARA version 11.3.22, YASARA Biosciences, Wien, Austria) was used to calculate ligand or solvent charges. In all protein-substrate docking cases, grid maps with 30 × 30 × 30 points and a grid-point spacing of 0.375 Å was used to cover the area of lid 1 (51–57), lid 2 (148–167) and active sites at Ser-207, Asp-255, His-313. The final trajectory of protein molecular dynamic in the presence of toluene or water was used as a protein template (in pdb format) and docked into respective substrates (alcohol, hexanoic acid and ethyl hexanoate). The grid map for latter docking-complex was 40 × 40 × 40 points due to the enlargement of lipase structure after simulation [33].

Each selected ligand or substrate was docked into the targeted binding sites one at a time. The docking was subjected to 25 runs with each docking sorted according to the strongest binding energy (kcal/mol). Then it carries out conformational cluster analysis on the docked conformations to determine which are similar, reporting the clusters ranked by increasing energy. At the end of each docking, AUTODOCK reports the dissociation constant, the coordinates of the docked conformation, and also the estimated free energy of binding. Images were generated by PyMOL (Educational-use-only, https://pymol.org/edu/?q=educational/)

### 3.4. Electrostatic Potential and Hydrophobic Effect Determination

Electrostatic potential was measured by Adaptive Poisson-Boltzmann Solver (APBS) and PDB2PQR software packages (http://www.poissonboltzmann.org/). The protein structure (in PDB) was made ready by using PDB2PQR software followed by calculation of electrostatic and apolar force outputs that were subjected for entire molecule based on a PBE equation. Other running parameters such as dielectric constant of solvent, grid-points per square angstrom, radius of solvent molecules and temperature were fixed as default. Water molecules were removed during the calculations and visualizations. The hydrophobic properties of molecules and their match or mismatch in receptor-ligand complexes were estimated on the basis of “Molecular Hydrophobic Potential” (MHP) via web-based analysis tool called Protein-Ligand ATtractions Investigation NUMerically (PLATINUM).

### 3.5. Kinetic Properties of AMS8 Lipase and Its Mutant

There were two types of quantitative lipase assays. For lipase purifications and initial organic solvent tolerance determination, all fractions containing lipase activity are determined by a simple and rapid colorimetric method from Kwon and Rhee [34]. Whereas, for kinetic analysis of purified AMS8 and L208A in the presence of polar and non-polar organic solvents are to be determined by the hydrolysis of pNP (*p*-nitrophenol) in 96-well plates [35]. The periodic measurement of the liberated *p*-nitrophenol (pNP) was based on the absorbance at 405 nm determined using a multiplate reader.

The hydrolytic activity of AMS8 and its mutant was measured by spectrophotometric method using Kwon and Rhee method [34]. Olive oil is the substrate of choice due to its oleic acid content determination in later quantification and the measurement was performed at an end-point (30 min of incubation) right after the reaction termination at 715 nm of spectrophotometrical absorbance. Enzyme kinetics was measured using *p*-nitrophenyl caproate (pNP-C8), laurate (pNP-C12) and palmitate (C16) as substrates. The reaction was started by adding ten microliters of pNP mixture in buffer of desired pH value. The enzyme-buffer mixture was pre-warmed at desired temperature before ten microliter of substrate was added to start the reaction. The absorbance of the reaction mixture was measured at 405 nm. One unit of enzyme activity is defined as the rate of release of 1 µmole of pNP per minute under the assay conditions.

### 3.6. Esterification

The modified procedure for esterification by Musa was used in this study [36]. The substrates (3.12 mL of 25 mM of hexanoic acid and 1.46 mL of 25 mM of ethanol) were mixed in 10 mL of the solvent which was toluene. This process was carried out in screw-capped universal bottles. Then, 0.15 g of molecular sieves was added immediately into the mixture. After that, 5 mg protein of crude freeze-dried lipase was added into the mixture. The mixture without lipases acted as the control of the process. The esterification process was done in 200 rpm water bath shaker at 20 °C or 30 °C for two hours. After incubation, the reaction mixtures were terminated using mixture of acetone and ethanol, ratio (1:1). The mixtures then were filtered by Whatmann No. 1 filter paper. Lastly, three drops of phenolphthalein was added and titrated with 0.5 M of NaOH using a burette by an acid-base titration method. This reaction was done in triplicate. The point at which the indicator changes colour is called the end point. When the end point close to the equivalence point of the reaction, the solution should be let out of the burette until the pH indicator which is phenolphthalein change to pink colour (alkaline) and the volume of NaOH on the burette was recorded. The conversion of ester was expressed as percentage conversion (%) under various reaction conditions according to the following formula:

$$\mathrm{Conversion}\;\mathrm{of}\;\mathrm{ester}\left(\%\right)=\frac{\left[\mathrm{volume}\;\mathrm{of}\;\mathrm{NaOH}\left(\mathrm{control}-\mathrm{with}\;\mathrm{enzyme}\right)\right]\times 100}{\mathrm{volume}\;\mathrm{NaOH}\left(\mathrm{control}\right)}$$

## 4. Conclusions

Disruption of molecular interactions which brings about a shorter range of distance between solvent-accessible residue L208A and the rest of catalytic triads affects the accessibility of bigger-sized and longer-chain substrates. The weakening of hydrogen bonding occurred between Ser-238 and Gly-210 in mutant L208A contributes to the collapse of the substrate binding site by distorting the shape of active sites and limits the access of various numbers and type of substrates. The stronger energy binding between substrate ethyl hexanoate with L208A mutant showed that the binding affinity was significantly increased contributing to lower K_M_ (when using caprylate) value as compared to its wild-type lipase, AMS8. There was no indication that lid activation occurred during the course of hydrolysis which promptly put this lipase in an inactive state unlike AMS8 lipase under similar conditions. Toluene in this case did not trigger structural movements of the lid (residue 148–157) through surface interaction while all active sites showed a rigid-like position that prevented full substrate accessibility. It has been hypothesized that the shorter lid 1 located at residue 51–57, which was stimulated by higher temperature of 35 °C could assist in supporting the catalytic function. Apparently, deterring the stability of lid 1 was not likely to encourage the synthesis of low-molecular weight esterification at higher temperature, 30 °C in L208A mutant where the rate of esterification plunged from 17.4% to 12.7%. Only WT AMS8 lipase exhibited a slight increase of esterification rate prior to temperature hikes. The present study demonstrates a 360° change of L208A lipase as it turns to be less tolerant in 25–30% (*v*/*v*) toluene, experiencing a lack in active site flexibility, reduced stability at moderate temperature, loss of thermolability features following simulation in toluene and a lowering of the rates of hydrolysis and esterification. More importantly, the lid activation was negated by minimizing the flexibility of the active site region. Overall, the outcome of a single-point mutation at Leu-208 can be a good indicator to relate significant relationships of the protein stability-flexibility-activity of cold-active AMS8 lipase.

## Figures and Tables

**Table 1:** A comparison of binding energy and number of clusters accumulated from all productive poses between AMS8 lipase and mutant L208A.

System	Substrate	Maximum Binding Energy (kcal/mol)	Number of Clusters	Contacting Residues (Cut Off: 5 Å Distance)
AMS8	Ethyl hexanoate	4.79	5	Leu-166, Gly-167, Asp-170, Tyr-171, Ala-172, Lys-173, Leu-211, Asn-214, Ser-215, Asp-218, Thr-240, Gln-241, Ser-242, Ser-243, Ser-264
	Ethanol	3.59	2	Phe-18, Ala-21, Met-22, Thr-25, Ser-204, Gly-205, Val-234, Ala-235 Tyr-236, Ile-250, Tyr-318
	Hexanoic acid	5.49	4	Tyr-29, His-30, Asn-31, Leu-32, Ser-60, Thr-61, Ser-63, Gln-64, Gly-65 Trp-72, Ser-76, Glu-77, Arg-106, Arg-141
L208A	Ethyl hexanoate	5.38	9	His-291, Trp-297, Asn-298, Pro-301, Phe-302, Ser-303, Asn-306, Pro-308, Thr-309, Asn-361
	Ethanol	3.52	3	Asp-255, Pro-256, Val-257, Phe-258, Arg-259, Ala-260, Ser-269, Ser-270, Gly-272,Val-273, His-274 Phe-302
	Hexanoic acid	5.97	4	Tyr-29, His-30, Leu-32, Ser-60, Thr-61, Ser-63, Gln-64, Gly-65, Trp-72 Ser-76, Glu-77, Arg-106, Gln-120 Arg-141

**Table 2:** A comparison of binding energy and number of clusters between AMS8 lipase and mutant L208A in complex system of lipase/toluene/substrate.

System	Substrate/Product	Maximum Binding Energy (kcal/mol)	Number of Clusters	Contacting Residues (Cut Off: 5 Å Distance)
AMS8	Ethyl hexanoate	5.56	7	His-30, Asn-31, Thr-61, Asp-62, Ser-63, Gln-64, Gly-65, Val-66, Ile-67, Ile-70, Trp-72, Glu-77, Lys-78
	Ethanol	3.43	5	Leu-184,Met-216, Leu-219, Ser-220, Lys-223, Trp-224, Ser-225, Gly-226, Phe-227, Tyr-228, Lys-229
	Hexanoic acid	5.50	6	Phe-140, Leu-184, Val-203, Met-216, Leu-219, Ser-220, Gly-221, Asn-222, Lys-223, Trp-224, Gly-226, Phe-227, Tyr-228
L208A	Ethyl hexanoate	5.65	7	Pro-50, Ala-51, Leu-53, Val-54, Ser-154, Ile-155, Gly-156, Asp-157, Val-257, Ile-304, Val-35, Asn-306, Val-307, Trp-310
	Ethanol	3.45	4	Ala-51, Ser-154, Ile-155,Gly-156, Asp-157, Ile-304, Val-305, Asn-306, Val-307
	Hexanoic acid	6.12	6	Ala-51, Ser-154, Ile-155, Gly-156, Asp-157, Pro-256, Val-257, Ile-304, Val-305, Asn-306, Val-307, Thr-309, Trp-310

**Table 3:** Mutant L208A substrate preference.

		Natural Oil	Mono-Esters		
		Olive Oil (umole/min/mg)	pNP Caprylate; C-8 (uM/min/mg)	pNP Laurate; C-12 (uM/min/mg)	pNP Palmitate; C-16 (uM/min/mg)
25–30% Toluene	L208A	0.187	0.253	0.132	0.301
	AMS8	0.751	0.731	0.041	0.687
0% Toluene	L208A	0.326	0.422	0.062	0.306
	AMS8	0.640	0.392	0.026	0.215

**Table 4:** Michaelis-Menten kinetics measuring K_M_ and K_cat_ of mutant L208A.

	AMS8 (Wild-Type)		L208A (Mutant)	
	Free Solvent	With Solvent	Free Solvent	With Solvent
K_M_ (µM)	A: 2.59 ± 2.7	A: 0.72 ± 0.1	A: 37.73 ± 22.3	A: 38.95 ± 5.9
	B: 1.85 ± 2.9	B: 0.69 ± 0.3	B: 1.11 ± 1.3	B: 32.28 ± 10.9
	C: 0.73 ± 2.0	C: 0.63 ± 0.7	C: 1.63 × 10^−16^ ± 0.0	C: 0.78 ± 0.5
Vmax	A: 1.84 × 10^−4^	A: 6.18 × 10^−5^	A: 1.67 × 10^−2^	A: 3.8 × 10^−4^
	B: 1.59 × 10^−4^	B: 6.31 × 10^−5^	B: 4.54 × 10^−4^	B: 1.75 × 10^−4^
	C: 2.44 × 10^−4^	C: 2.18 × 10^−4^	C: 1.10 × 10^−3^	C: 1.16 × 10^−4^
K_cat_ (s^−1^)	A: 2.16 × 10^−7^	A: 5.02 × 10^−6^	A: 5.47 × 10^−3^	A: 3.16 × 10^−5^
	B: 1.86 × 10^−7^	B: 5.13 × 10^−6^	B: 1.49 × 10^−4^	B: 1.42 × 10^−5^
	C: 2.87 × 10^−7^	C: 1.97 × 10^−5^	C: 3.61 × 10^−4^	C: 5.38 × 10^−6^
K_cat_/K_M_ (µM^−1^s^−1^)	A: 8.37 × 10^−8^	A: 6.92 × 10^−6^	A: 1.45 × 10^−4^	A: 8.10 × 10^−7^
	B: 1.01 × 10^−7^	B: 7.44 × 10^−6^	B: 1.34 × 10^−4^	B: 4.41 × 10^−7^
	C: 3.93 × 10^−7^	C: 1.56 × 10^−4^	C: 2.22 × 10^12^	C: 1.01 × 10^−5^

Substrates: A is for pNP palmitate (C-16), B is for pNP laurate (C-12) and C is for pNP caprylate (C-8) Notes: K_cat_ = a direct measure of the catalytic production of product under optimum conditions (saturated point); K_cat_/K_M_ = Enzyme efficiency determined by the frequency with which enzyme and substrate molecules can collide.

## Supplementary Materials

The following are available online. Figure S1: Superimposed L208A lipase at 0 and 20 nanoseconds of simulation in toluene at 35 °C. Figure S2: Formation of a “tunnel” in the middle of AMS8 protein’s core providing access for substrates to bind at catalytic binding sites. Figure S3: The root-mean-square deviations (RMSd and RMSf) of mutant L208A simulated with ester, ethyl hexanoate at different temperature (20 °C and 30 °C). Figure S4: The radius of gyration, Rg and secondary structures of mutant L208A simulated with ester, ethyl hexanoate at different temperature (20 °C and 30 °C).Table S1: Electrostatic interaction and Molecular Hydrophobic Potential (MHP) between substrate ethyl hexanoate with L208A and WT lipases in the presence of water and toluene.
