# Supplementary material for: The Role of Solvent-Accessible Leu-208 of Cold-Active Pseudomonas fluorescens Strain AMS8 Lipase in Interfacial Activation, Substrate Accessibility and Low-Molecular Weight Esterification in the Presence of Toluene
Source: Molecules. 2017 Aug 12;22(8):1312. doi: 10.3390/molecules22081312 (PMC6152135; doi:10.3390/molecules22081312)
Supplement: Supplementary file 1 [file molecules-22-01312-s001.pdf]

Supplementary Table

Table S1. Electrostatic interaction and Molecular Hydrophobic Potential (MHP) between substrate ethyl hexanoate with L208A and WT lipases in the presence of water and toluene.

	L208A					WT				
	**kJ/mol**	**H-bonds**	**$S_{H/H}$, Å**	**$[2S_{L/L} /(2S_{L/L} + S_{HL} + S_{LH} + S_{LW}]$**	**K_{cat} (s^{-1})**	**kJ/mol**	**H-bonds**	**$S_{H/H}$, Å**	**$[2S_{L/L} /(2S_{L/L} + S_{HL} + S_{LH} + S_{LW}]$**	**K_{cat} (s^{-1})**
Water /25°C	287.81	0.0	0.38	0.69	3.61×10^{-4}	1205.33	0.51	3.16	0.14	2.87×10^{-7}
Toluene /25°C	1205.21	1.17	3.16	0.380	5.38×10^{-6}	1021.44	0.0	3.16	0.0	1.97×10^{-5}

Notes: kJ/mol: Electrostatic energy: $S_{H/H}$, Å : Area of hydrophilic match surface; $[2S_{L/L} /(2S_{L/L} + S_{HL} + S_{LH} + S_{LW}]$: Fraction of matching hydrophobic surface

Supplementary Figures

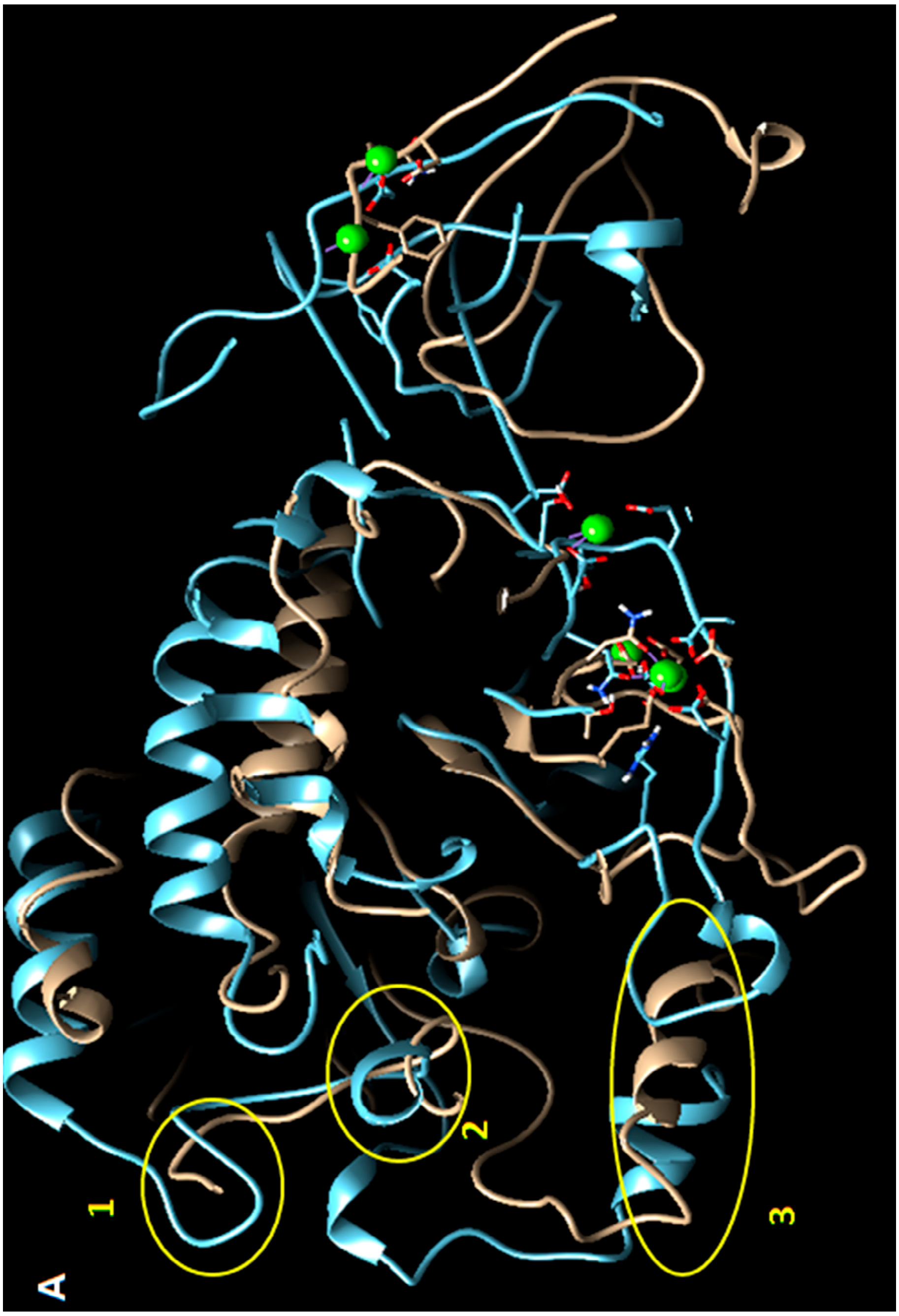

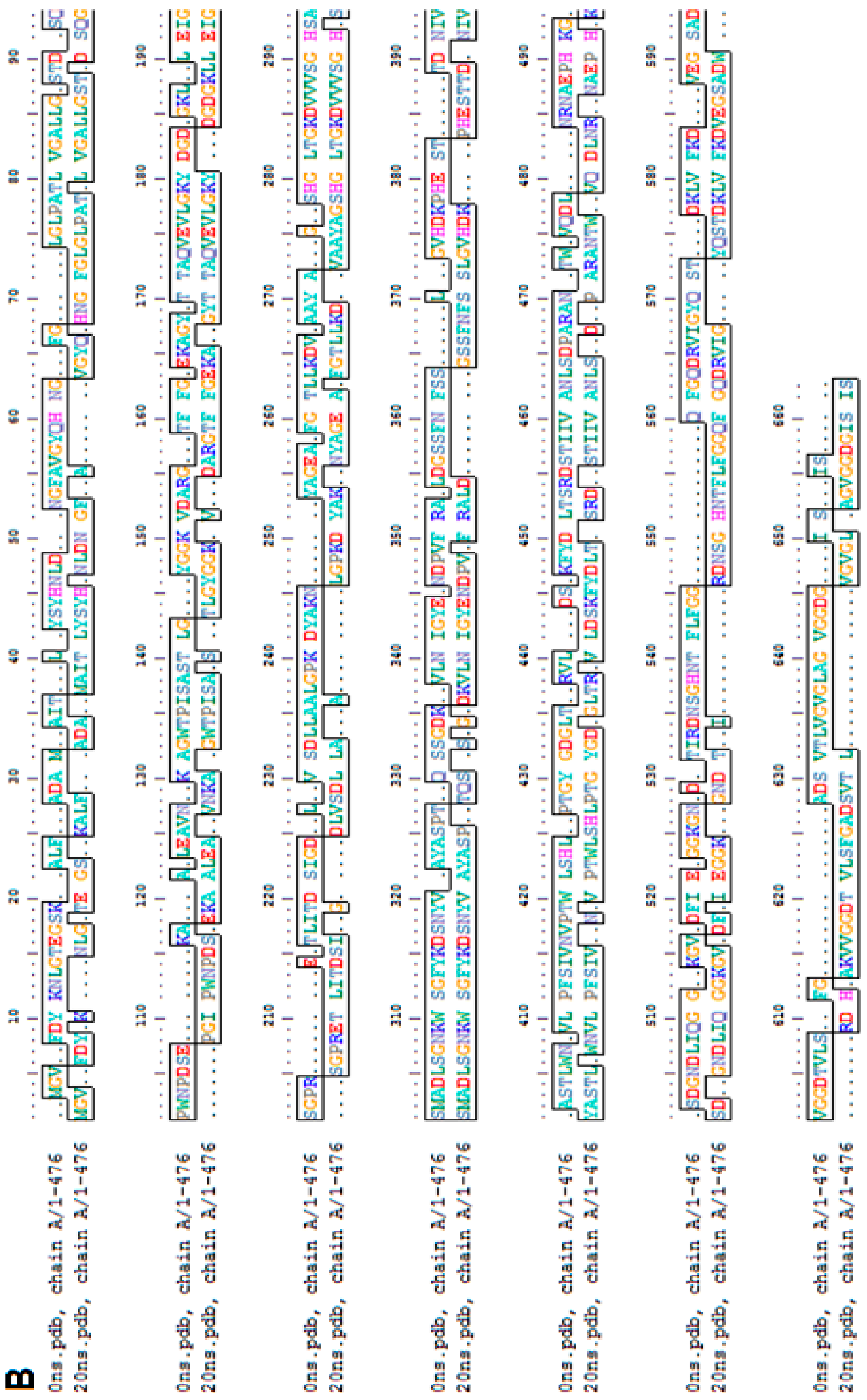

Figure S1. Superimposed L208A lipase at 0 and 20 nanoseconds of simulation in toluene at 35 °C. The three fluctuated sites highlighting residues (1) Ala-51 to Leu-57, (2) Ser-60 to Gly-65 and (3) Glu-148 to Gly-167 were identified in circle (image A) where the grey and blue- coloured ribbon represents L208A lipase at 0 ns and 20 ns respectively and the clustal alignment of superimposed protein sequences was observed having a match of 31.72% with RMSd 10.54 Å over 6932 matched atom (B). The superimpose analysis was performed using UCSF Chimera version 1.10.1

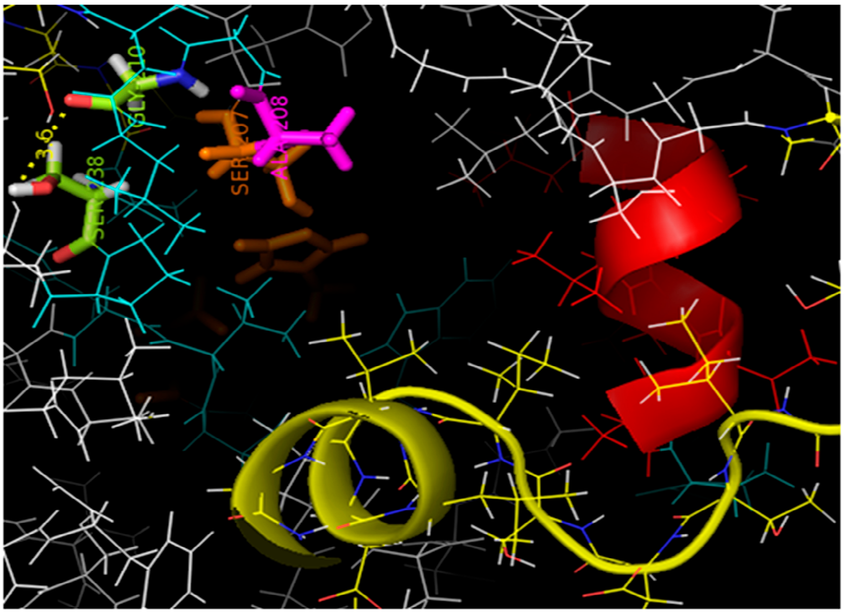

B

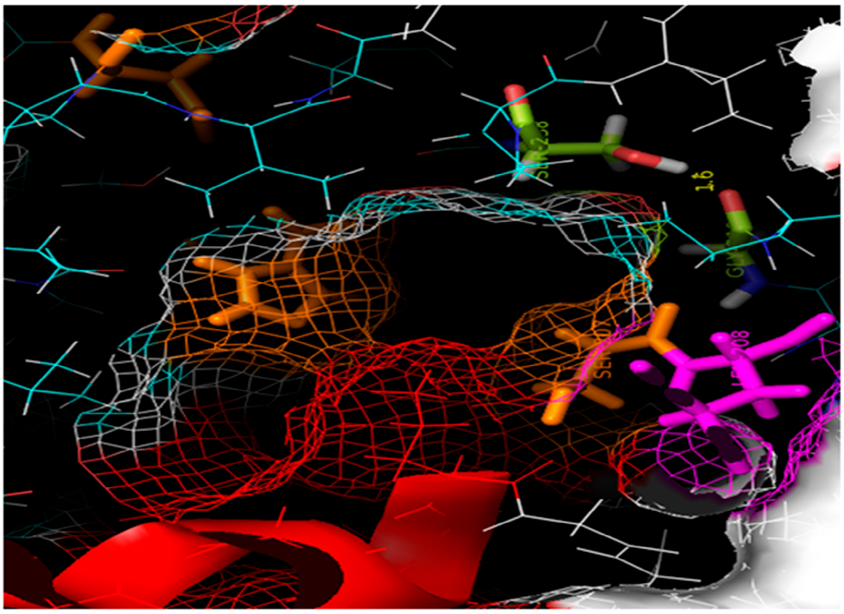

A

Figure S2. Formation of a “tunnel” in the middle of AMS8 protein’s core providing access for substrates to bind at catalytic binding sites (orange sticks). A funnel-like binding site such as WT AMS8 lipase simulated in toluene was formed showing the formation of hydrogen bonds (1.6 Å) between Ser-238 and Gly-210 (A). However, the collapse of funnel-shaped binding site with an altered hydrogen bond formation (3.6 Å) between similar residues in mutant L208A indicates the major geometrical changes which cannot be repaired with the presence of toluene (B). Lid 1 is indicated in red helix, lid 2 in yellow helix, residue L208/A208 is identified in magenta stick and hydrogen bond forming residues Ser-238 and Gly-210 are colored based on group elements showing the distance between oxygen from Ser-238 and hydroxyl groups from Gly-210.

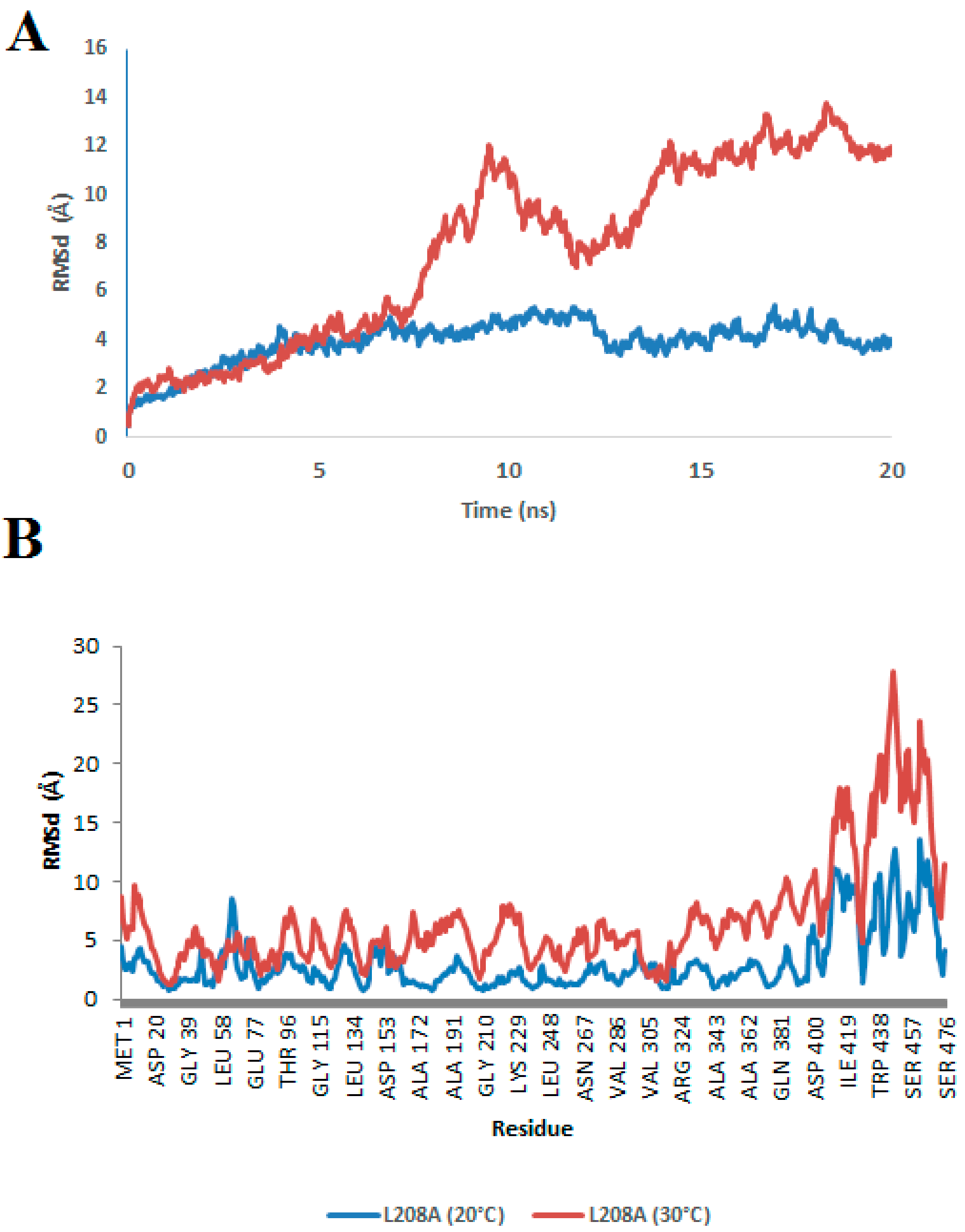

Figure S3. The root-mean-square, RMSd (A) and RMSf (B) of mutant L208A simulated with ester, ethyl hexanoate at different temperature (20°C and 30°C)

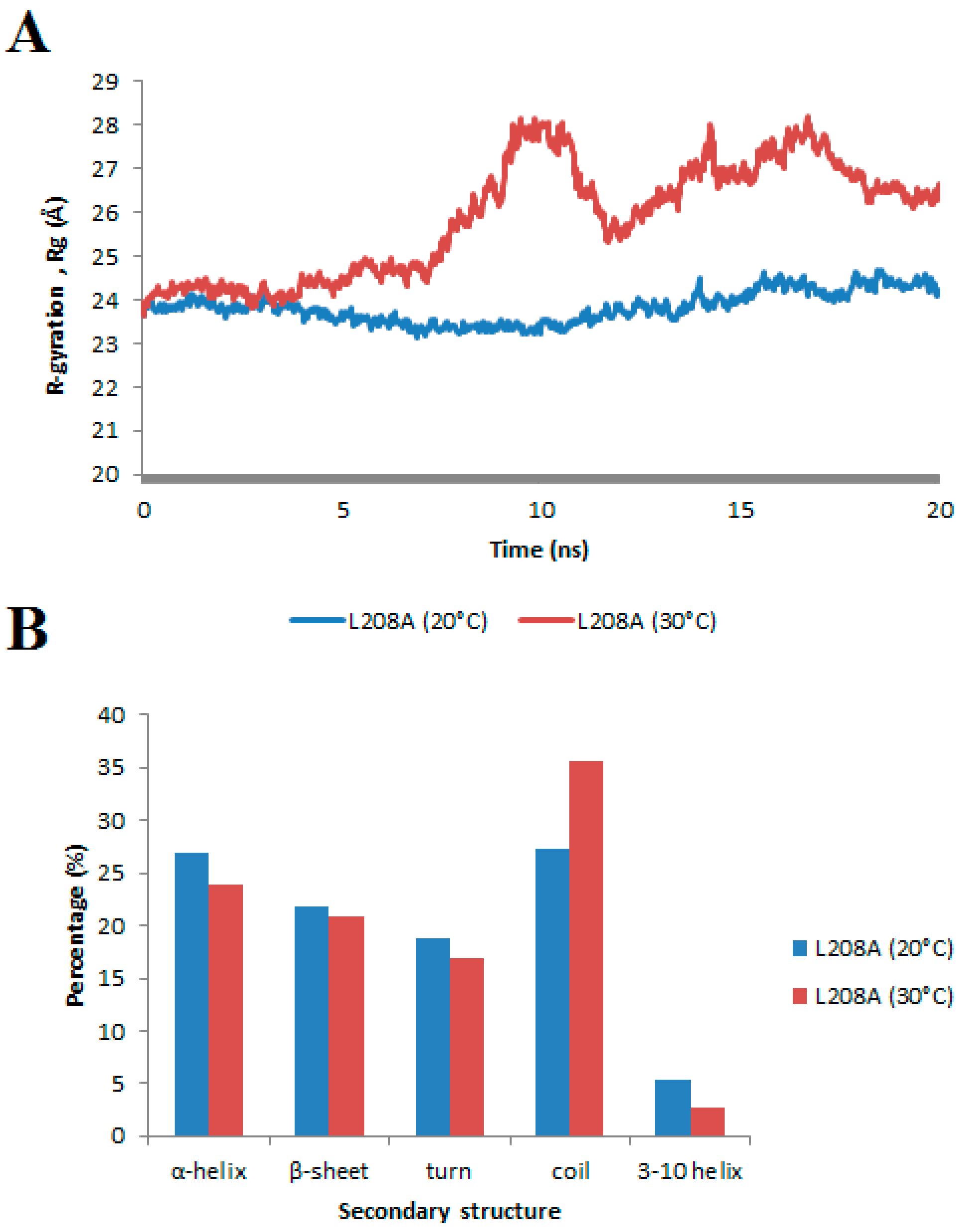

Figure S4. The radius of gyration, Rg (A) and secondary structures (B) of mutant L208A simulated with ester, ethyl hexanoate at different temperature (20°C and 30°C)
